# Arginase-1 promotes lens epithelial-to-mesenchymal transition in different models of anterior subcapsular cataract

**DOI:** 10.1186/s12964-023-01210-4

**Published:** 2023-09-18

**Authors:** Qingyu Li, Yuchuan Wang, Luoluo Shi, Qing Wang, Guang Yang, Lin Deng, Ye Tian, Xia Hua, Xiaoyong Yuan

**Affiliations:** 1 Department of Cataract, Tianjin Eye Hospital, Tianjin, China; 2 Tianjin Key Lab of Ophthalmology and Visual Science, Tianjin, China; 3 Clinical College of Ophthalmology, Tianjin Medical University, Tianjin, China; 4 Heze Medical College, Heze, Shandong China; 5 School of Microelectronics, Tianjin University, Tianjin, China; 6 Tianjin Aier Eye Hospital, Tianjin University, Tianjin, China

**Keywords:** ARG1, Anterior subcapsular cataract, Posterior capsular opacification, Arginase-related pathway, CB-1158

## Abstract

**Background:**

Arginase-1 (ARG1) promotes collagen synthesis and cell proliferation. ARG1 is highly expressed in various tumour cells. The mechanisms of ARG1 in epithelial-to-mesenchymal transition (EMT)-associated cataracts were studied herein.

**Methods:**

C57BL/6 mice, a human lens epithelial cell line (HLEC-SRA01/04), and human lens capsule samples were used in this study. The right lens anterior capsule of the mouse eye was punctured through the central cornea with a 26-gauge hypodermic needle. Human lens epithelial cells (HLECs) were transfected with ARG1-targeted (siARG1) or negative control siRNA (siNC). For gene overexpression, HLECs were transfected with a plasmid bearing the ARG1 coding sequence or an empty vector. Medium containing 0.2% serum with or without transforming growth factor beta-2 (TGF-β2) was added for 6 or 24 h to detect mRNA or protein, respectively. The expression of related genes was measured by quantitative real-time polymerase chain reaction (RT–qPCR), western blotting, and immunohistochemical staining. Transwell assays and wound healing assays were used to determine cell migration. Cell proliferation, superoxide levels, nitric oxide (NO) levels, and arginase activity were estimated using Cell Counting Kit-8 assays, a superoxide assay kit, an NO assay kit, and an arginase activity kit.

**Results:**

ARG1, alpha-smooth muscle actin (α-SMA), fibronectin, and Ki67 expression increased after lens capsular injury, while zonula occludens-1 (ZO-1) expression decreased.

Fibronectin and collagen type I alpha1 chain (collagen 1A1) expression increased, and cell migration increased significantly in ARG1-overexpressing HLECs compared with those transfected with an empty vector after TGF-β2 treatment. These effects were reversed by ARG1 knockdown.

The arginase-related pathway plays an important role in EMT. mRNAs of enzymes of the arginase-related pathway were highly expressed after ARG1 overexpression. ARG1 knockdown suppressed these expression changes.

Numidargistat (CB-1158) dihydrochloride (CB-1158), an ARG1 inhibitor, suppressed TGF-β2-induced anterior subcapsular cataract (ASC) by reducing the proliferation of lens epithelial cells (LECs) and decreasing fibronectin, α-SMA, collagen 1A1, and vimentin expression.

Compared with that in nonanterior subcapsular cataract (non-ASC) patients, the expression of ARG1, collagen 1A1, vimentin, fibronectin, and Ki67 was markedly increased in ASC patients.

**Conclusions:**

ARG1 can regulate EMT in EMT-associated cataracts. Based on the pathogenesis of ASC, these findings are expected to provide new therapeutic strategies for patients.

**Graphical abstract:**

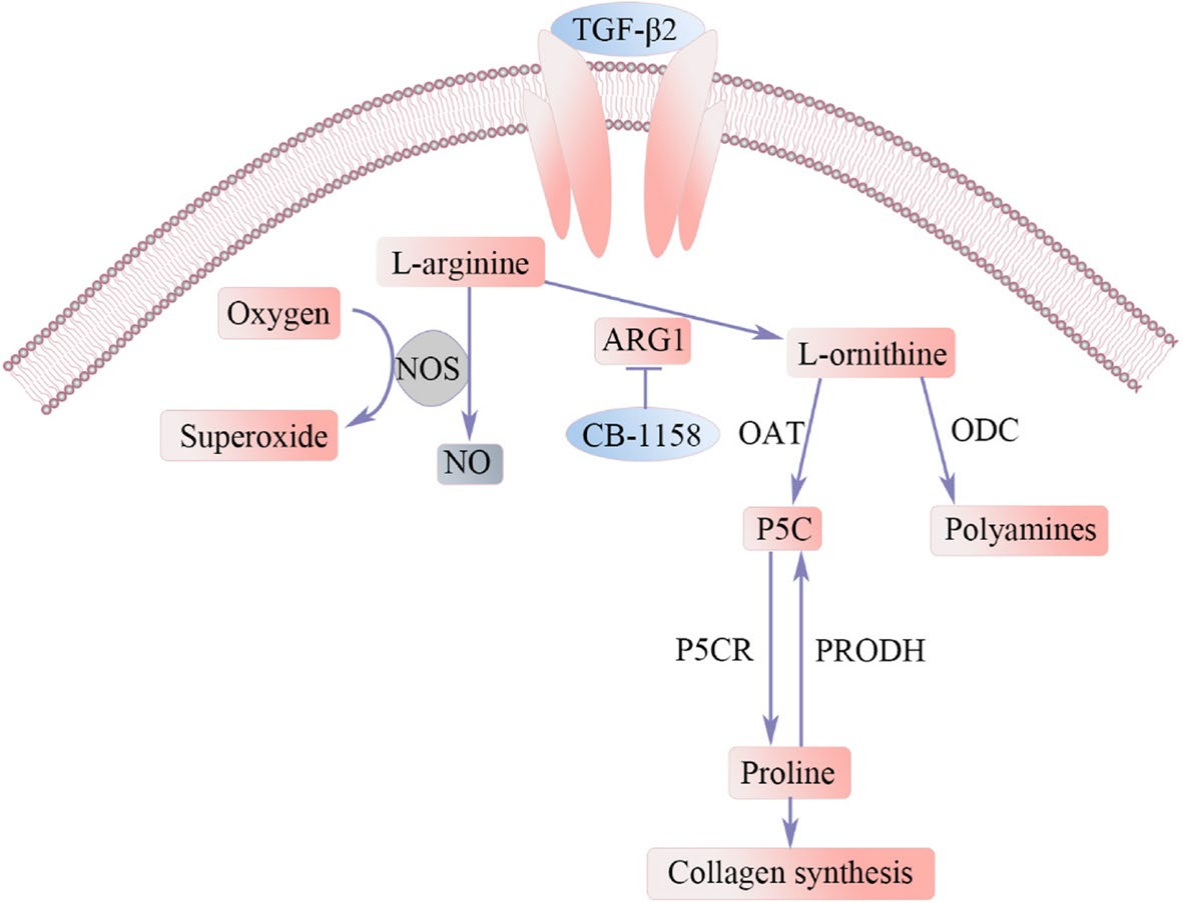

**Supplementary Information:**

The online version contains supplementary material available at 10.1186/s12964-023-01210-4.

## Abstract (plain language summary)

Fibrotic cataracts can be classified as anterior subcapsular cataract or posterior capsular opacification depending on where fibrosis occurs. The mechanism of fibrotic cataracts is not fully understood. Fibrotic opacities induced by trauma, inflammation, or radiation can accumulate underneath the anterior lens capsule, causing anterior subcapsular cataract. Posterior capsular opacification is one of the most common complications of phacoemulsification with intraocular lens implantation, with a high incidence in young patients. We show for the first time that ARG1 can regulate EMT in fibrotic cataracts. TGF-β2 is the main cause of fibrosis in LECs. The expression of ARG1 and fibronectin in LECs increased after TGF-β2 treatment or mouse lens capsular injury. We investigated the specific molecular mechanisms by which ARG1 regulates EMT in fibrotic cataracts. The mRNA expression of enzymes of the arginase-related pathway was decreased due to knockdown of ARG1 expression in HLECs. These effects were reversed by ARG1 overexpression. Additionally, knockdown of ARG1 decreased collagen 1A1, fibronectin, and vimentin expression; superoxide levels; and cell migration and increased NO levels. These effects were reversed by ARG1 overexpression. Pharmacological blockade of the ARG1 pathway with CB-1158 reduced the proliferation of LECs and decreased fibronectin, α-SMA, collagen 1A1, and vimentin expression in mouse lenses. We believe that ARG1 promotes the production of collagen 1A1 by directly activating the arginase pathway and leads to lens fibrosis by reducing NO production and increasing superoxide levels, providing a new mechanism for the prevention and treatment of fibrotic cataracts.

Video Abstract

**Supplementary Information:**

The online version contains supplementary material available at 10.1186/s12964-023-01210-4.

## Background

Fibrotic cataracts can be classified as ASC or posterior capsular opacification (PCO) depending on where fibrosis occurs. ASC and PCO share many cellular and molecular features [1, 2]. Fibrotic opacities induced by trauma, inflammation, or radiation can accumulate underneath the anterior lens capsule, causing ASC [3]. PCO is one of the most common complications of phacoemulsification with intraocular lens (IOL) implantation [4], mostly occurring soon to a few years after cataract surgery [5, 6], with a high incidence in young patients [7]. After cataract surgery, residual LECs in the anterior and equatorial regions proliferate, migrate, transform into a myofibroblastic phenotype and secrete excessive extracellular matrix proteins on the posterior capsule, leading to PCO [8, 9]. Neodymium-doped yttrium aluminium garnet (Nd:YAG) capsulotomy must be performed to remove the fibrous tissue and restore vision in such patients. The mechanism of EMT-associated cataracts is not fully understood. The anterior chamber aqueous humour has been shown to contain significant amounts of TGF-β2 [10]. TGF-β2 is the main cause of fibrosis in LECs [11]. Once TGF-β2 is activated, LECs secrete excess extracellular matrix components, including collagen 1A1, collagen type IV (COL4), and fibronectin [12]. Moreover, due to downregulation of the epithelial marker ZO-1, cell polarity is lost, and the adhesion between cells is weakened. LECs acquire apolar, migratory, and myofibroblastic features by synthesizing α-SMA and vimentin [3]. A previous study examined the genetic changes in the remaining LECs after cataract surgery in mice and showed a 411-fold increase in ARG1 expression in the remaining LECs compared with that of the control group [13].

Arginase is a binuclear manganese-containing metalloenzyme in the urea cycle that hydrolyses L-arginine to generate urea and ornithine, which are further metabolized to polyamines and proline [14]. L-ornithine is a substrate for the synthesis of polyamines by ornithine decarboxylase (ODC). The starting substrate L-ornithine is first converted to pyrroline-5- carboxylate (P5C) by ornithine aminotransferase (OAT) and subsequently reduced to proline by P5C reductase (P5CR). Proline dehydrogenase (PRODH) degrades proline into P5C. Polyamines can cause cell proliferation through the regulation of gene expression and are highly expressed in tumour cells [15–18]. Proline is a precursor for collagen synthesis. Fibrotic opacities in the lens are characterized by increased production and deposition of extracellular matrix components, particularly collagen, and enhanced proliferation and migration of myofibroblastic LECs [1, 19]. In lens fibrosis, L-arginine metabolism is altered by the expression and activity of ARG1, which ultimately affects collagen synthesis. Exogenous NO may retard the development of fibrosis by preventing oxidative damage-induced EMT [20, 21]. Arginase activation leads to decreased NO bioavailability, increased superoxide levels, and decreased antifibrotic effects [22–25]. For inhibition of ARG1, 50 μM CB-1158 [26] was added to the medium to prevent TGF-β2-induced ASC formation for 7 days.

To elucidate the regulatory roles of ARG1 in the development of EMT-associated cataracts, we established a mouse anterior lens capsule injury model, a TGF-β2-induced EMT model and a mouse lens culture model and used human anterior capsule membrane samples.

## Methods

### Ethics statement

The adult male C57BL/6 mice used in this study were purchased from Jinan Pengyue Experimental Animal Breeding Co., Ltd., (Jianan, China) and raised in the Experimental Animal Center of Nankai Hospital. All procedures involving animals were conducted strictly in accordance with the Association for Research in Vision and Ophthalmology (ARVO) Statement for the use of Animals in Ophthalmic and Vision Research. All animal experiments were formally reviewed and approved by the Animal Care and Ethics Committee of the Nankai Hospital (Approval number: NKYY-DWLL-2022–088). The study involving human subjects followed the tenets of the Declaration of Helsinki and was formally reviewed and approved by the Tianjin Eye Hospital Medical Ethics Committee. Informed consent was obtained from patients before the collection of human lens capsular tissues. Human ASC lens capsule specimens were collected from patients diagnosed with ASC. The non-ASC group was nuclear or cortical cataract patients with clear lens capsules and no other ocular disease. After capsulorhexis during cataract surgery, tissue samples of the anterior capsular membranes were collected.

### Mouse lens capsular injury model

The mice were anaesthetized with an intraperitoneal injection of pentobarbital sodium (40–50 mg/kg), and a drop of proparacaine was applied to the right corneal surface. The right lens anterior capsule of the mouse eye was punctured through the central cornea with a 26-gauge hypodermic needle, as described previously [27]. The depth of puncture was approximately one-fourth of the length of the blade part of the 26-gauge needle. The mice were sacrificed, and the eyes were enucleated for immunohistochemistry on the seventh day after the injury.

### Lens culture and treatment

The lenses of 21-day-old mice were used as described previously [28]. Briefly, the lenses were carefully removed using forceps and kept in M199 medium containing 0.1% BSA (# PM150610A, Pricella, Wuhan, China). TGF-β2 and CB-1158 (#HY-101979A, MedChemExpress, New Jersey, USA) were added to the medium at final concentrations of 10 ng/ml and 50 μM, respectively. The medium was changed every other day. After 7 days of lens culture, photographs were taken using a dissecting microscope.

### Cell culture

HLEC-SRA01/04 cells were purchased from Saiku Biotechnology Co. Ltd. (#CC4022, Guangzhou, China), and cultured in a humidified atmosphere of 5% CO_2_ at 37 °C. HLECs were cultured in Dulbecco’s modified Eagle’s medium (DMEM) containing 20% foetal bovine serum (FBS) (Gibco, Grand Island, New York, USA). When the HLECs reached 80% confluence, the cells were seeded in six-well plates. Serum-free medium was added overnight before TGF-β2 (R&D Systems, Minnesota, USA) treatment. Medium containing 0.2% serum with or without TGF-β2 was added to the wells. For gene silencing, HLECs were transfected with siARG1 or siNC when the cell confluence reached 60–80% using Advanced DNA RNA Transfection Reagent (#AD600025, Zeta Life, USA) according to the manufacturer’s instructions. Briefly, the Advanced DNA RNA Transfection Reagent and siRNA were mixed and incubated for 15 min, and then, the complex was added to the cells. After incubation for 24 h, the cells were treated with serum-free medium overnight. All siRNAs were purchased from Hanbio Co., Ltd. The target sequences for siARG1-1 were 5'-GGAAACAUCCGAUAUAAAUCUTT -3' (sense) and 5'-AGAUUUAUAUCGGAUGUUUCCTT-3' (antisense). The target sequences for siARG1-2 were 5'-GGAGACAAAGCUACCACAUGUTT-3' (sense) and 5'-ACAUGUGGUAGCUUUGUCUCCTT-3' (antisense). The target sequences for siARG1-3 were 5'-GAGUUAUCCUUCUAAAGACUUTT-3' (sense) and 5'-AAGUCUUUAGAAGGAUAACUCTT-3' (antisense). The target sequences for siNC were 5'-UUCUCCGAACGUGUCACGUTT-3' (sense) and 5'-ACGUGACACGUUCGGAGAATT-3' (antisense). Medium containing 0.2% serum with or without TGF-β2 was then added to the wells for 6 or 24 h to detect mRNA or protein, respectively. For gene overexpression, HLECs were transfected with a plasmid bearing the ARG1 coding sequence or an empty vector. The transfection procedure was the same as described above.

### RT–qPCR

Cells in one well of a six-well plate were prepared for each group. RNA was extracted with EZB-RN001-plus (EZBioscience, Roseville, MN, USA) according to the kit instructions. After the RNA concentration was measured using a Nanodrop 2000 system (Thermo, Boston, USA), 1 μg RNA was used to synthesize cDNA with Uni All-in-One Supermix and gDNA remover (TransGen Biotech, Beijing, China). The expression of ARG1, fibronectin, ZO-1, vimentin, collagen 1A1, P5CR, PRODH, ODC, and OAT was measured by using SYBR Green qPCR Supermix (TransGen Biotech, Beijing, China). The threshold cycle number for each mRNA was normalized to that of glyceraldehyde-3-phosphate dehydrogenase (GAPDH) mRNA and averaged. Each experiment was independently repeated three times. The following primer pairs were used: for ARG1, 5'- TGGACAGACTAGGAATTGGCA-3' and 5'-CCAGTCCGTCAACATCAAAACT-3'; for fibronectin, 5'-GAGCTGCACATGTCTTGGGAAC-3' and 5'- GGAGCAAATGGCACCGAGATA-3'; for collagen 1A1, 5'- GAGGGCCAAGACGAAGACATC-3' and 5'-CAGATCACGTCATCGCACAAC-3'; for ZO-1, 5'-ACCAGTAAGTCGTCCTGATCC-3' and 5'- TCGGCCAAATCTTCTCACTCC-3'; for vimentin, 5'- ATTCCACTTTGCGTTCAAGG-3' and 5'-CTTCAGAGAGAGGAAGCCGA-3'; for PRODH, 5'-TCTGTTGCTGTCTTCACGGA-3' and 5'- CCTGGAAACATACAGCAGCCTAT-3'; for ODC, 5'- CTGGGCGCTCTGAGATTGTC-3' and 5'-AGCAAGGGTCTTCACGATGG-3'; for OAT, 5'-AGGCGCTGTCAGATCTGTGG-3' and 5'- CTCCGCGACTAAGTACAGCA-3'; for P5CR, 5'- AGCTCCATTGAGAAGAAGCTGT-3' and 5'- CATCTTGGCAGCCCCGTA-3'; and for GAPDH, 5'-GAGTCAACGGATTTGGTCGT-3' and 5'- AATGAAGGGGTCATTGATGG-3'.

### Western blotting

Cells in one well of a six-well plate were prepared for each group. Cells were lysed using RIPA lysis buffer (Beyotime, Shanghai, China) containing protease inhibitor (TransGen Biotech, Beijing, China). After agarose gel electrophoresis, fibronectin, ZO-1, collagen 1A1, vimentin, ARG1, and β-actin were detected using the primary antibodies rabbit anti-fibronectin (1:500; ABclonal, Wuhan, China), rabbit anti-ZO-1 (1:500; ABclonal), rabbit anti-ARG1 (1:500; ABclonal), mouse anti-vimentin (1:500; Beyotime), rabbit anti-collagen 1A1 (1:500; Huabio, Hangzhou, China), and mouse anti-actin (1:1000; TransGen Biotech, Beijing, China). The PVDF membrane was then incubated with secondary antibodies for 1 h at room temperature. After the membrane was washed, the protein bands were detected using ECL chemiluminescence solution (Beyotime). The grey value for each protein was normalized to that of β-actin and averaged. Each experiment was independently repeated three times.

### Haematoxylin and eosin (H&E) and immunohistochemical staining

Human lens capsular tissues from patients with ASC were obtained during cataract surgery. Age-matched lens capsular tissues from patients with nuclear or cortical cataracts were included in this study as controls. Seven days post-anterior capsule puncture, the mice were sacrificed, and their eyeballs were isolated for H&E and immunohistochemical staining. The lenses of mice treated with TGF-β2 and CB-1158 were used in this study. The enucleated eyes, human lens capsular tissues, and mouse lenses were fixed with 4% paraformaldehyde overnight and then embedded in paraffin. Four-micron-thick slices were used for H&E and immunohistochemical staining. The samples were then stained with H&E. Immunohistochemistry was performed using primary antibodies against ARG1, ZO-1, fibronectin, α-SMA, vimentin, Ki67, and collagen 1A1 (1:100; ABclonal). Three human lens capsular tissues were used for each group in the study. The number of Ki67-positive cells in a specific area was detected using image j software. Finally, the Student’s t test was used for statistics.

### Transwell assay

The cells in each group were digested with trypsin, centrifuged at 1000 rpm for 5 min, and then washed twice with PBS. The cells were resuspended in medium with or without TGF-β2 and counted on cell counting plates. Twenty-four-well Transwell plates with 8-μm pores were used in our experiments. One hundred microlitres of serum-free medium was first added to the upper chamber, followed by 200 μl of cells (2.5 × 10^5^ cells/ml). Then, 750 μl of complete medium was added to the lower chamber. After 16 h, the upper chamber was fixed with paraformaldehyde for 10 min and then stained with crystal violet for 30 min. A cotton swab was used to clean the cells on the upper surface of the chamber. Four pictures of each inferior surface of the upper chamber were taken with a Nikon ECLIPSE Ti system (Nikon, Minato-ku, Tokyo, Japan). Each experiment was repeated three times.

### Wound healing assay

The rate of wound closure was observed using 2-well silicone inserts (ibidi, Gräfelfing, Germany). The HLEC suspension was adjusted to a cell concentration of 5 × 10^5^ cells/ml. Seventy microlitres of cell suspension was added to each well. The cells were cultured overnight at 37 °C with 5% CO_2_. The 2-well inserts were gently removed with sterile forceps and washed twice with PBS. Cell medium with or without TGF-β2 was added to each well. Photographs were taken every 12 h for a total of 48 h using a Cytation™ 5 Cell Imaging Multi-Mode Reader (BioTek, Vermont, USA).

### Arginase activity

HLECs were treated with TGF-β2 for 0, 6, 12, 24, or 48 h. Whole lysates of HLECs from different groups were prepared for evaluation with an arginase activity kit (#ARG050, Lablead, Beijing, China). The test reagents were added according to the manufacturer’s protocols. The optical density (OD) was measured at 430 nm using a microplate reader (BioTek, Vermont, USA).

### Cell Counting Kit-8 (CCK-8) assay

Cell proliferation was estimated using CCK-8 assays (#C0037, Beyotime, Shanghai, China). Cells were seeded in 96-well plates at a density of 4 × 10^4^ cells/ml. After the cells were fully adherent, 10 µl of CCK-8 solution was added to each well. The OD was measured at 450 nm after 2, 24, and 48 h using a microplate reader (BioTek, Vermont, USA).

### Superoxide assay kit

Superoxide levels were estimated using a superoxide assay kit (#S0060, Beyotime, Shanghai, China). Cells were seeded in 96-well plates at a density of 5 × 10^4^ cells/ml. After the cells were fully adherent, 200 µl of superoxide assay solution with or without TGF-β2 was added to each well. The OD was measured at 450 nm after 1 h using a microplate reader (BioTek, Vermont, USA).

### In situ NO assay

NO was detected using 3-amino,4-aminomethyl-2',7'-difluorescein, diacetate (DAF-FM DA) (#S0019, Beyotime, Shanghai, China). After treatment with TGF-β2 for 24 h, HLECs were incubated with DAF-FM DA in a humidified atmosphere at 37 °C for 20 min. Next, the HLECs were rinsed twice with PBS. The fluorescence intensity of DAF-FM DA was measured using a Cytation™ 5 Cell Imaging Multi-Mode Reader (BioTek, Vermont, USA).

### Statistical analysis

All experiments were performed in triplicate. Data are expressed as the means ± SDs. One-way ANOVA was used for comparisons among more than two groups. Student’s t test was used to evaluate differences between two groups. All calculations and statistical tests were analysed using GraphPad Prism 7.0 (GraphPad Software, San Diego, CA, USA). A P value of less than 0.05 was considered significant.

## Results

### ARG1 expression is substantially increased in the ASC model

**Fig. 1:**
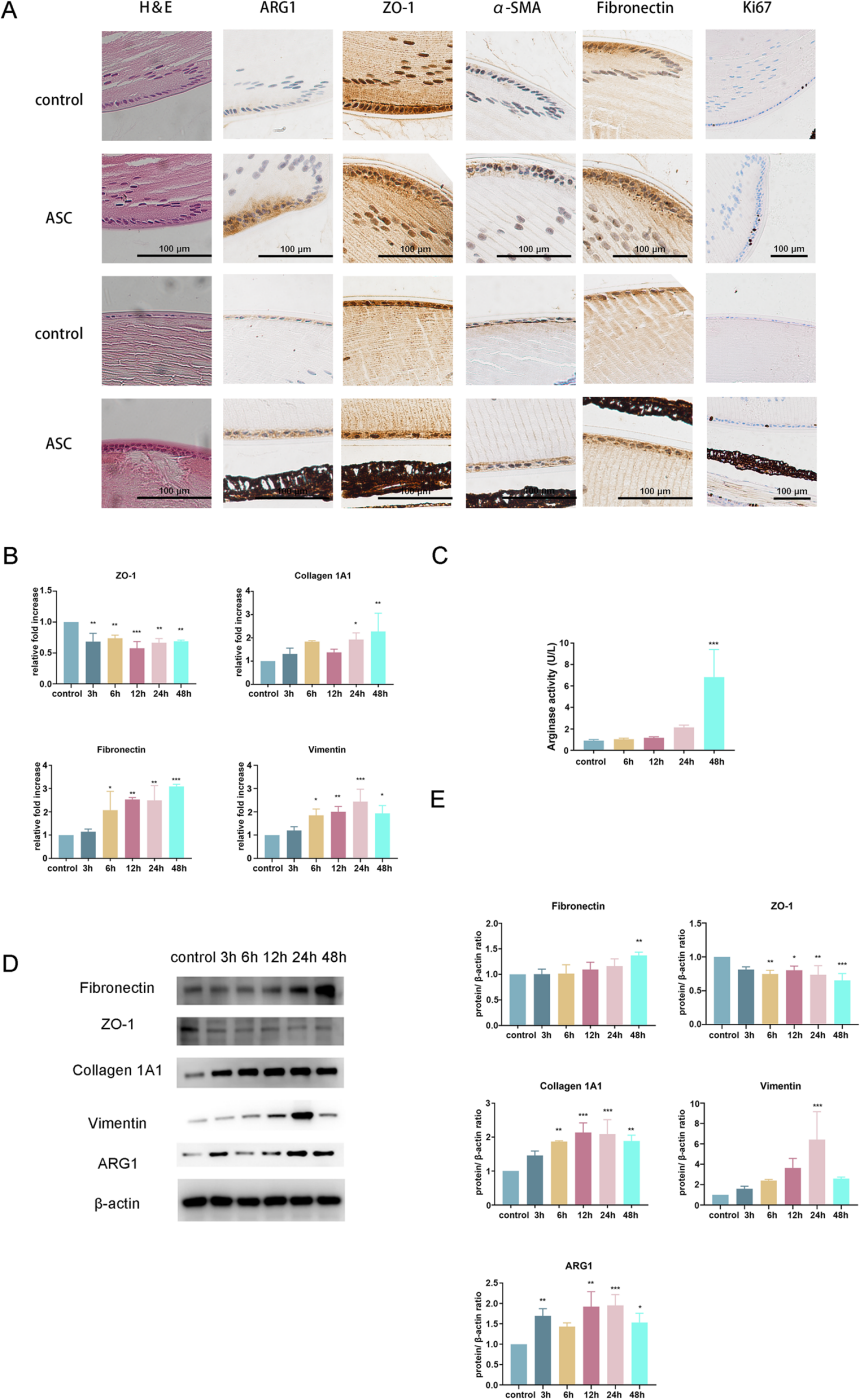
ARG1 expression is markedly increased after TGF-β2 treatment. **A** Representative images of H&E and immunohistochemical staining of the punctured area and equatorial regions of the lens capsule after ASC injury. **B** RT–qPCR analysis demonstrating significantly downregulated expression of ZO-1 and upregulated expression of collagen 1A1, fibronectin, and vimentin in HLECs treated with TGF-β2 (*N* = three). **C** Arginase activity was increased substantially 48 h after TGF-β2 treatment. **D** Western blot analysis demonstrating significantly upregulated expression of fibronectin, collagen 1A1, vimentin, and ARG1 and downregulated expression of ZO-1 in HLECs treated with TGF-β2 (*N* = three). The error bars represent the means ± SDs, and comparisons were made using one-way ANOVA. ASC, anterior subcapsular cataract; H&E, haematoxylin and eosin staining; ARG1, arginase-1; ZO-1, zonula occludens-1. *P < 0.05, **P < 0.01, and ***P < 0.001

First, we established a murine ASC model. Lens epithelial cells release a large number of biological mediators after injury, which promote gene expression changes in equatorial cells. Previous studies have found high expression of immediate early genes at both the wounded regions and the equatorial regions after injury in rat lens epithelial cells [29]. After ocular trauma, the proliferation and migration of LECs in situ and equatorial lens epithelial cells lead to the formation of subcapsular plaques [27, 30]. H&E staining showed that LECs developed ASC with disorganized proliferation (Fig. 1A). Immunohistochemical staining revealed notably high expression of ARG1 and the mesenchymal markers α-SMA, fibronectin, and Ki67 and concomitant low expression of the epithelial marker ZO-1 in the murine ASC model (Fig. 1A).

RT–qPCR and western blot analysis revealed significantly upregulated expression of fibronectin, collagen 1A1, and the mesenchymal marker vimentin and downregulated expression of ZO-1 in HLECs after TGF-β2 treatment (Fig. 1B, D, E). Western blot analysis revealed significantly upregulated expression of ARG1 in HLECs after TGF-β2 treatment (Fig. 1D, E). Arginase activity was increased substantially 48 h after TGF-β2 treatment (Fig. 1C). These results suggest that the upregulation of ARG1 due to TGF-β2 activation promotes the progression of EMT.

### ARG1 knockdown decreases collagen 1A1, fibronectin, and vimentin expression and cell migration

**Fig. 2:**
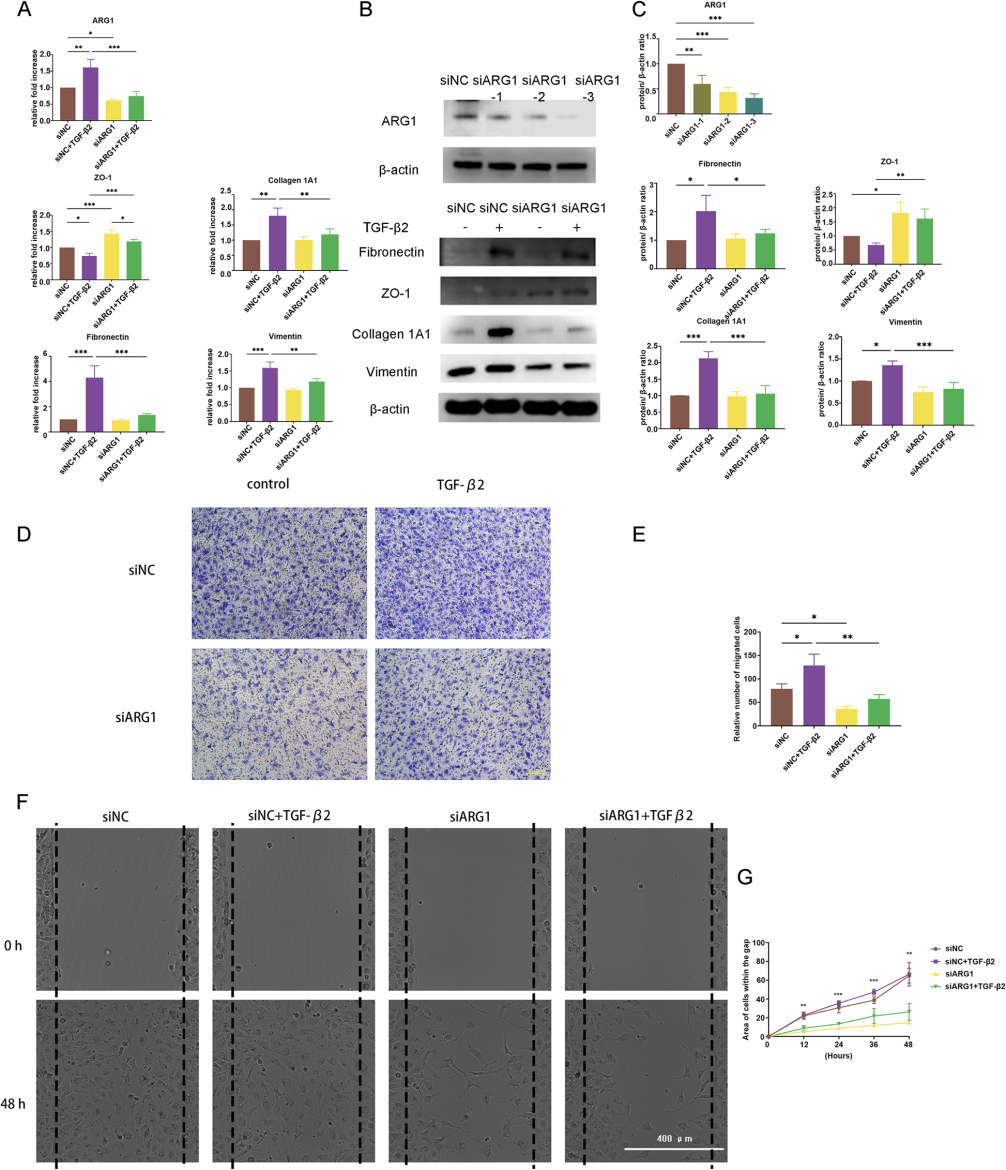
ARG1 knockdown decreases collagen 1A1, fibronectin, and vimentin expression and cell migration. **A** RT–qPCR analysis of ARG1 gene knockdown efficiency. The mRNA expression levels of ZO-1, collagen 1A1, fibronectin, and vimentin in HLECs treated with or without TGF-β2 after ARG1 knockdown were detected by RT–qPCR (*N* = three). **B**, **C** The protein expression levels and quantification of ARG1, fibronectin, ZO-1, collagen 1A1, and vimentin in HLECs treated with or without TGF-β2 after ARG1 knockdown were detected by western blot analysis (*N* = three). **D**, **E** Representative images and quantification of HLECs on the inferior surface of the upper chamber following a Transwell assay after ARG1 knockdown (scale bar: 100 μm). **F**, **G** Representative images and quantification of the migration of HLECs transfected with siARG1 (scale bar: 400 μm). The statistical results only showed the difference between siNC + TGF-β2 group and siARG1 + TGF-β2 group. Error bars represent the mean ± SD, and comparisons were performed using one-way ANOVA. *P < 0.05, **P < 0.01, and ***P < 0.001

To examine the role of ARG1 in EMT, we compared cell migration in response to TGF-β2 treatment in HLECs transfected with siARG1 or siNC. The knockdown efficiency of ARG1 was verified by RT–qPCR and western blotting, which showed that ARG1 was significantly downregulated in the HLECs transfected with siARG1 compared to the HLECs transfected with siNC (Fig. 2A, B, C). We chose siARG1-3 for ARG1 knockdown in the subsequent experiments.

ARG1 knockdown significantly reduced the increase in fibronectin, collagen 1A1 and vimentin expression induced by TGF-β2 treatment at both the mRNA and protein levels (Fig. 2A, B, C). ARG1 knockdown significantly restored the reduction in ZO-1 expression induced by TGF-β2 treatment at both the protein and mRNA levels (Fig. 2A, B, C).

Transwell assays and wound healing assays revealed that the HLECs transfected with siARG1 exhibited notably decreased cell migration compared with those transfected with siNC after TGF-β2 treatment (Fig. 2D, E, F, G). These results suggest that siARG1 controls the migration of HLECs, reprogramming HLECs towards a physiological phenotype.

### ARG1 overexpression increases collagen 1A1, fibronectin, and vimentin expression and cell migration

**Fig. 3:**
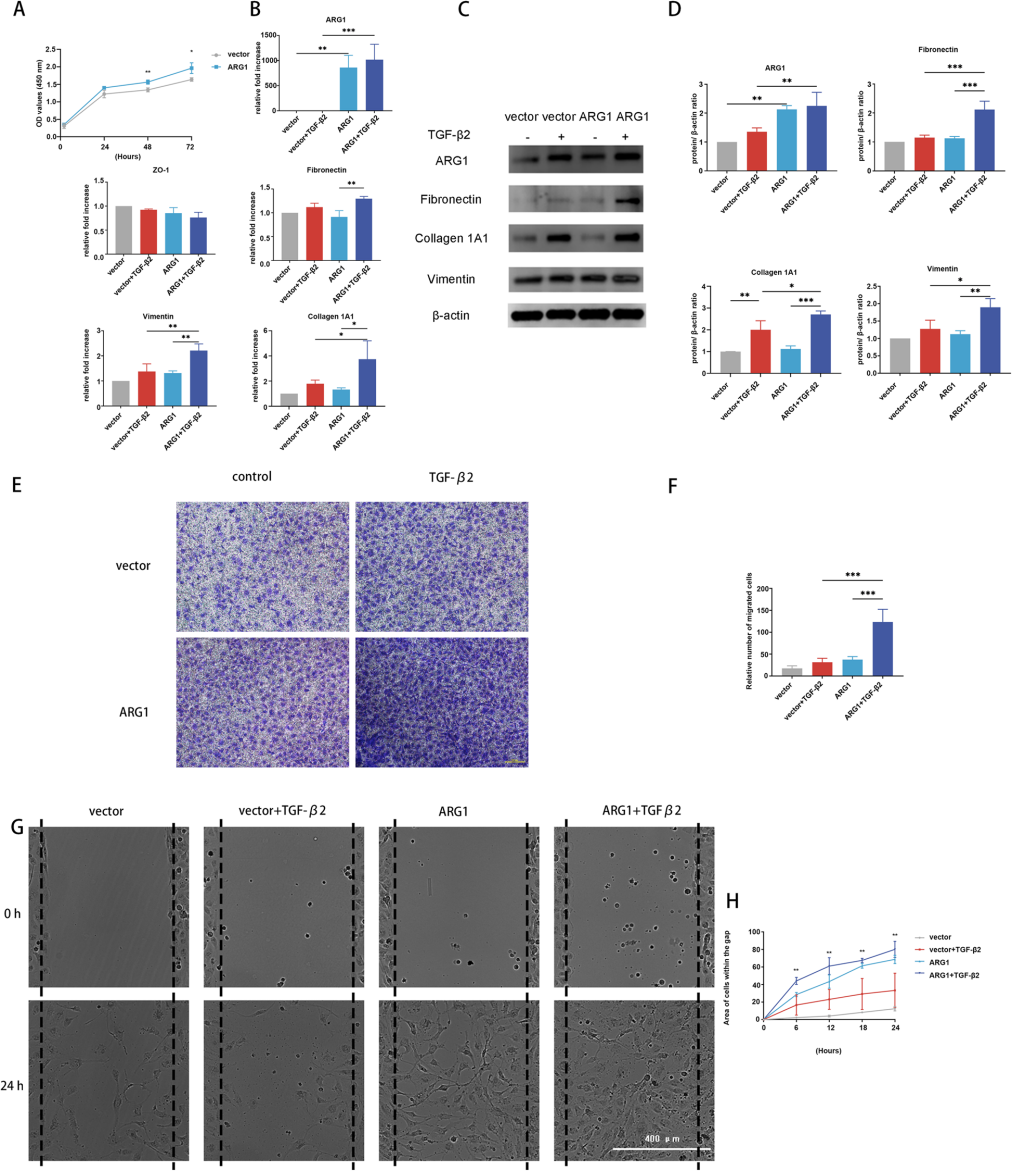
ARG1 overexpression increases collagen 1A1, fibronectin, and vimentin expression and cell migration. **A** Proliferative activity of HLECs after ARG1 overexpression. **B** The mRNA expression levels of ARG1, ZO-1, collagen 1A1, fibronectin, and vimentin in HLECs treated with or without TGF-β2 after ARG1 overexpression were detected by RT–qPCR (*N* = three). **C**, **D** The protein expression levels and quantification of ARG1, fibronectin, collagen 1A1, and vimentin in HLECs treated with or without TGF-β2 after ARG1 overexpression were detected by western blot analysis (*N* = three). **E**, **F** Representative images and quantification of HLECs on the inferior surface of the upper chamber following a Transwell assay after ARG1 overexpression (scale bar: 100 μm). **G**, **H** Representative images and quantification of the migration of HLECs transfected with a plasmid bearing the ARG1 coding sequence (scale bar: 400 μm). The statistical results only showed the difference between vector + TGF-β2 group and ARG1 + TGF-β2 group. Error bars represent the mean ± SD, and comparisons were performed using one-way ANOVA and Student’s t test. *P < 0.05, **P < 0.01, and ***P < 0.001

To verify the role of ARG1 in EMT, we compared cell migration in response to TGF-β2 treatment in the HLECs transfected with a plasmid bearing the ARG1 coding sequence and the HLECs transfected with an empty vector. ARG1 gene overexpression was confirmed by RT–qPCR and western blotting, which showed that ARG1 was significantly upregulated in the HLECs transfected with a plasmid bearing the ARG1 coding sequence compared with the HLECs transfected with an empty vector (Fig. 3B, C, D). ARG1 overexpression significantly increased HLEC proliferation at 48 and 72 h (Fig. 3A).

ARG1 overexpression significantly enhanced the increase in collagen 1A1 and vimentin expression induced by TGF-β2 treatment at both the mRNA and protein levels (Fig. 3B, C, D). ARG1 overexpression increased the reduction in ZO-1 expression, but this difference was not significant (Fig. 3B). Furthermore, ARG1 overexpression did not significantly increase fibronectin mRNA expression but did increase fibronectin protein expression (Fig. 3B, C, D).

Transwell assays and wound healing assays revealed that ARG1 overexpression notably increased cell migration compared with that of the cells transfected with an empty vector after TGF-β2 treatment (Fig. 3E, F, G, H).

### The role of the arginase-related pathway in EMT

**Fig. 4:**
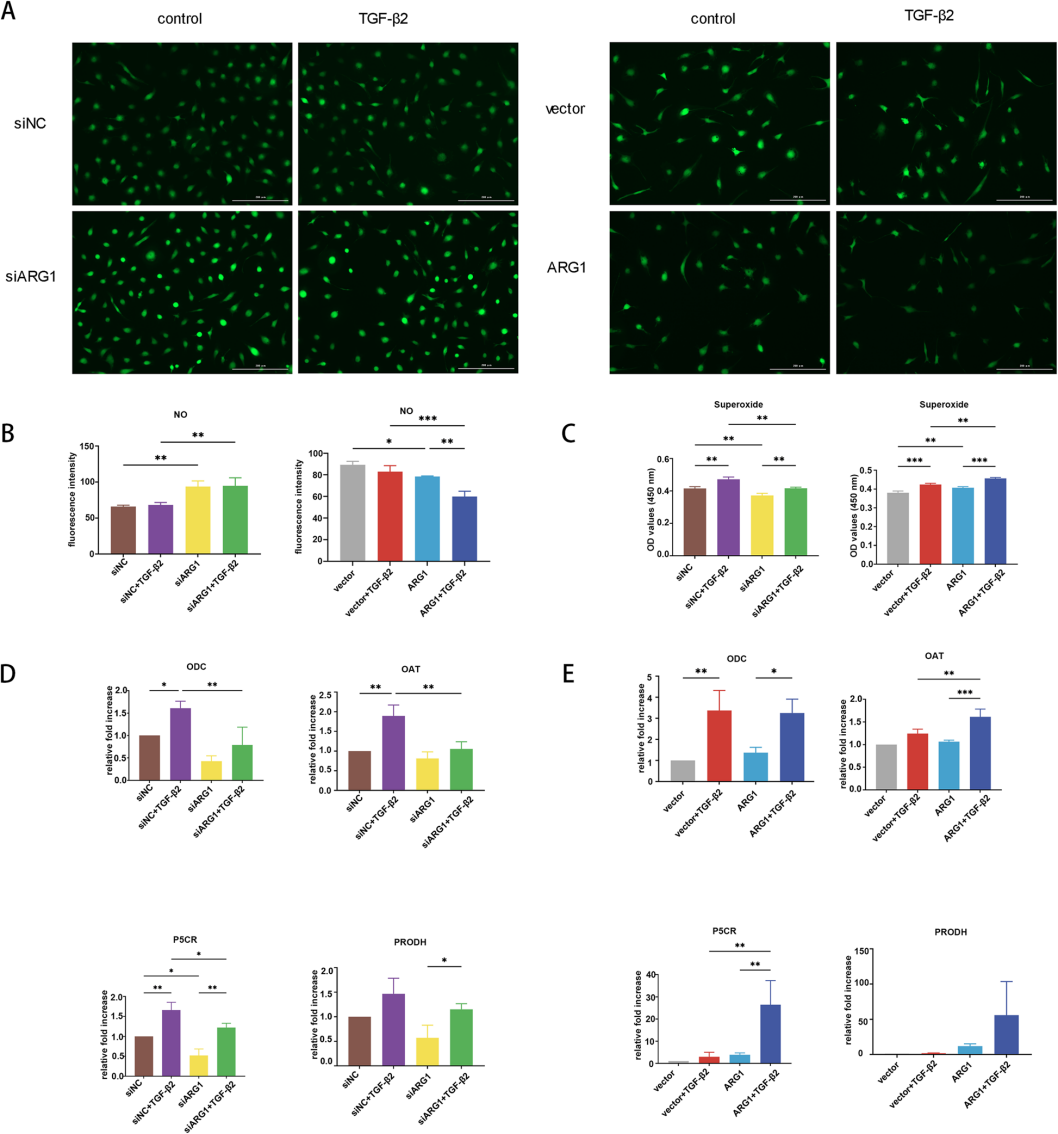
The role of the arginase-related pathway in EMT. **A**, **B** Representative images and quantification of the NO level in HLECs after ARG1 overexpression or knockdown (scale bar: 200 μm). **B** Quantification of the superoxide level in HLECs after ARG1 overexpression or knockdown. **D**, **E** The mRNA expression levels of ODC, OAT, P5CR, and PRODH after ARG1 overexpression or knockdown were detected by RT–qPCR (*N* = three). The error bars represent the mean ± SD, and comparisons were performed using one-way ANOVA. NO, nitric oxide; ODC, ornithine decarboxylase; OAT, ornithine aminotransferase; P5CR, pyrroline-5-carboxylate reductase; PRODH, proline dehydrogenase. *P < 0.05, **P < 0.01, and ***P < 0.001

ARG1 knockdown significantly increased, while ARG1 overexpression significantly decreased, the NO level (Fig. 4A, B). ARG1 knockdown significantly decreased the increase in superoxide levels induced by TGF-β2 treatment (Fig. 4C). ARG1 overexpression significantly increased the increase in superoxide levels induced by TGF-β2 treatment (Fig. 4C). To evaluate the profibrotic arginase-related pathway, we quantified the mRNA expression of enzymes of the arginase-related pathway. ARG1 knockdown significantly reduced the increase in ODC, OAT, and P5CR expression induced by TGF-β2 treatment at the mRNA level. ARG1 knockdown reduced the increase in PRODH expression, but this difference was not significant. To verify the role of ARG1 in the arginase-related pathway, we detected changes in the mRNA expression of these genes after ARG1 was overexpressed. ARG1 overexpression enhanced the increase in OAT and P5CR expression induced by TGF-β2 treatment at the mRNA level (Fig. 4E). ARG1 overexpression increased the elevation in PRODH expression, but this difference was not significant (Fig. 4E). ARG1 overexpression did not change the expression of ODC in HLECs (Fig. 4E).

### CB-1158, an ARG1 inhibitor, prevents TGF-β2-induced ASC

**Fig. 5:**
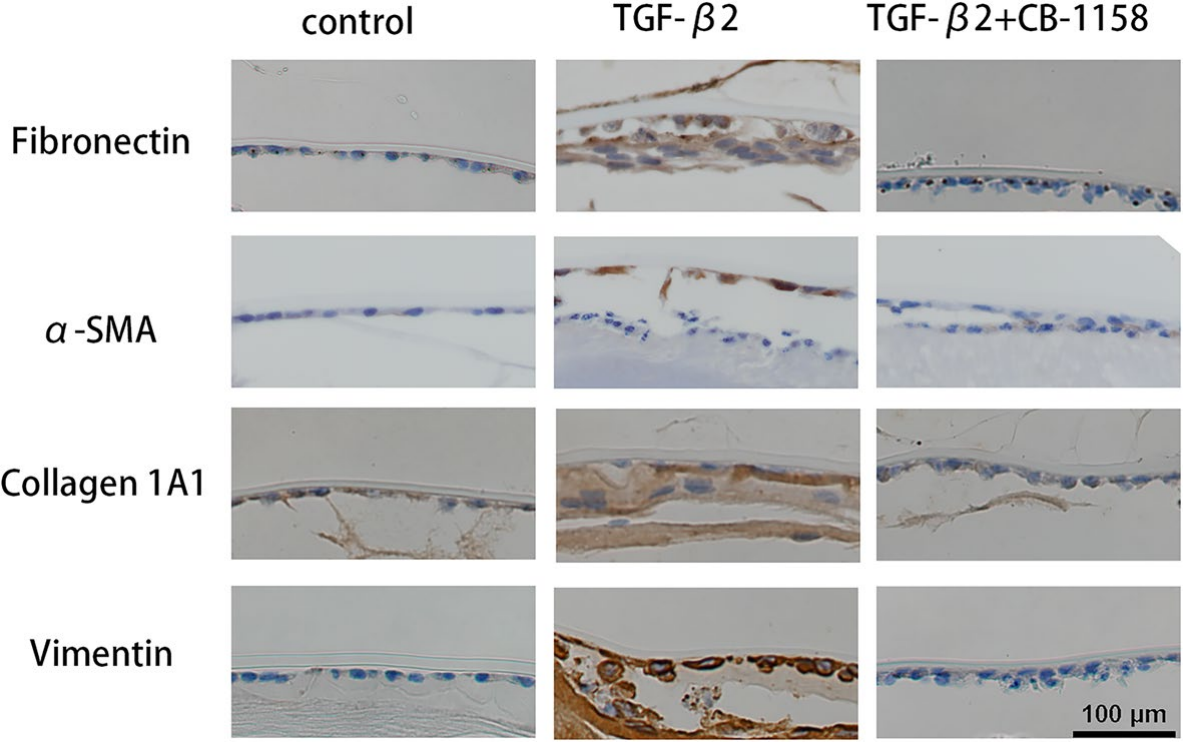
Decreased expression of fibronectin, vimentin, collagen 1A1, and Ki67 was verified in mouse lenses treated with CB-1158. **A**, **B** Representative photographs of mouse lenses and images of immunohistochemical staining of mouse lens capsular tissues treated with or without CB-1158

To determine whether inhibition of ARG1 can reduce mouse lens fibrosis, we cultured mouse lenses with CB-1158 and TGF-β2 for 7 days. In the TGF-β2 group, mouse lenses exhibited increased expression of fibronectin, α-SMA, collagen 1A1, and vimentin (Fig. 5). In contrast, CB-1158 suppressed TGF-β2-induced ASC. The CB-1158-treated lenses exhibited decreased fibronectin, α-SMA, collagen 1A1, and vimentin expression (Fig. 5). Taken together, these results suggest that ASC development can be suppressed by inhibiting ARG1.

### Increased expression of ARG1, fibronectin, vimentin, collagen 1A1, and Ki67 was verified in patients with ASC

**Fig. 6:**
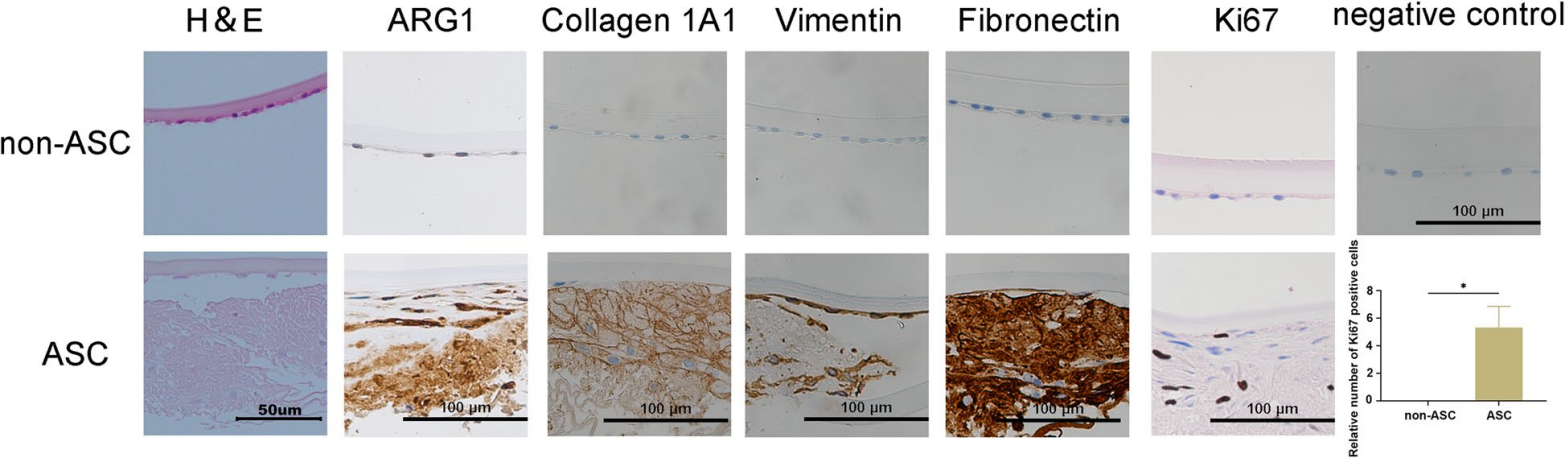
Increased expression of ARG1, fibronectin, vimentin, collagen 1A1, and Ki67 was verified in patients with ASC. Representative images of H&E and immunohistochemical staining of human lens capsular tissues in patients with or without ASC

We observed morphological changes in the lens epithelium, which changed from a single layer of cells to multiple layers of cells with a large amount of extracellular matrix deposition in patients with ASC. We further explored the expression of ARG1, collagen 1A1, vimentin, fibronectin, and Ki67 in human lens capsule samples from non-ASC patients and patients with ASC (Fig. 6). Compared with those observed in the non-ASC patients, the expression levels of ARG1, collagen 1A1, vimentin, fibronectin, and Ki67 in the patients with ASC were markedly increased (Fig. 6), which was consistent with our observations in murine models, indicating the involvement of ARG1 in ASC patients.

## Discussion

Fibrosis can occur in various organs [31–33]. Continuous progression can lead to the destruction and even failure of organ structures, which seriously threatens human health and life [34]. Lens fibrosis results in a loss of vision. Currently, the best treatment for ASC is surgery, and for PCO, it is Nd:YAG capsulotomy. Methods other than surgical treatment still need further study.

Tumour-associated fibrosis is an important component of the tumour microenvironment [35–37]. Fibrotic lesions, EMT, and cell proliferation contribute to tumour progression in various tissues [38]. The fibrosis of tumours is similar to that of the lens to some extent. Available data have demonstrated the abnormally high expression and activity of arginase in gastric cancer [39], breast cancer cells [40, 41], malignant skin tumours [42], and cervical cancer [43]. Tumours in ARG1 KO mice were 50% smaller than those in wild-type mice [44]. Topical administration of the arginase inhibitor Nω-hydroxy-nor-L-arginine monoacetate (nor-NOHA) decreased the growth of cutaneous squamous cell carcinoma [45]. ARG1 is the key enzyme in the urea cycle that hydrolyses L-arginine to generate urea and L-ornithine, which can supply precursor substrates for proline and polyamine production. Fibrotic opacities in the lens are characterized by increased deposition of extracellular matrix components, particularly collagen 1A1, and enhanced proliferation of myofibroblastic phenotype LECs. In lens fibrosis, L-arginine metabolism is altered by the expression and activity of ARG1, which ultimately affects collagen synthesis and the proliferation of LECs.

Subsequently, we further investigated the molecular mechanisms by which fibrosis is regulated by ARG1. ARG1 was highly expressed in HLECs treated with TGF-β2, injured mouse lens capsules and lens capsule samples from patients with ASC. ARG1 knockdown significantly reduced the increase in ODC, OAT, and P5CR expression induced by TGF-β2 treatment at the mRNA level. ARG1 knockdown significantly reduced the increase in collagen 1A1 expression induced by TGF-β2 treatment at both the mRNA and protein levels. ARG1 knockdown also notably decreased cell migration. The mRNA expression of OAT and P5CR was high in the ARG1-overexpressing HLECs treated with TGF-β2. ARG1 overexpression significantly increased HLEC proliferation after TGF-β2 treatment. It may be due to the fact that ARG1 is highly expressed in the cells of the control group. Compared with the control group, differences after ARG1 overexpression could not be detected by RT-qPCR and western blotting. When TGF-β2 was added, the ARG1 overexpression group showed significant differences compared with the control group. The mRNA and protein levels of collagen 1A1 were high in the ARG1-overexpressing HLECs treated with TGF-β2. ARG1 overexpression also notably increased cell migration after TGF-β2 treatment. ARG1 enhanced the proliferation and migration of myofibroblastic HLECs by regulating the expression of enzymes of the arginase-related pathway.

ARG1 competes with nitric oxide synthase (NOS) for L-arginine, not only decreasing NO formation but also increasing superoxide production by NOS via the one-electron reduction of oxygen [46–49]. When ARG1 activity is enhanced in tumour cells and leads to low L-arginine concentrations, the NOS reductase domain may generate superoxide [50–52]. NO has been shown to have antifibrotic properties. Inducible NOS (iNOS) and NO downregulation promoted EMT and metastasis in colorectal cancer [20]. An NO donor exhibited antitumour activity through inhibition of EMT in human lung cancer and melanoma cells [21]. Increased reactive oxygen species (ROS) levels lead to the development of fibrosis by inducing oxidative damage during EMT in the lens. ROS produced by different pathways promoted the development of both ASC and PCO in cultured lens epithelial explants [53]. Antioxidants reduced either glutathione or catalase and suppressed TGF-β-induced subcapsular plaque formation and opacification in cultured rat lenses and lens epithelial explants [54]. ARG1 knockdown attenuated TGF-β2-induced EMT by maintaining NO levels and decreasing superoxide levels. ARG1 overexpression led to decreased NO production, increased superoxide levels, and decreased antifibrotic effects. ARG1 may contribute to the progression of EMT in LECs by mediating L-arginine metabolism, polyamine synthesis, NO bioavailability and oxidative stress.

ARG1 inhibitors are now potential antitumour drugs [45, 55–57]. Blocking arginase activity pharmacologically with nor-NOHA can reduce the growth of various tumours [45]. CB-1158 is a potent small-molecule inhibitor of ARG1. CB-1158 was tested for its ability to inhibit arginase enzymes. CB-1158 could selectively inhibit ARG1 [55]. CB-1158 blocked myeloid cell-mediated suppression of T-cell proliferation in vitro [55]. CB-1158 as a single agent and in combination with standard-of-care chemotherapy or other immunotherapies inhibited tumour growth in mice [55]. CB-1158 is more likely to inhibit cytoplasmic or extracellular ARG1 in plasma, tumours, and inflamed tissues [55]. In human and mouse lens capsular samples, we found significant expression of ARG1 in the extracellular tissue. Here, we selected CB-1158 as an inhibitor of ARG1. CB-1158 blocked TGF-β2-induced ASC by reducing the proliferation of LECs and decreasing fibronectin, α-SMA, collagen 1A1, and vimentin expression. This study showed that blocking arginase activity with CB-1158 could reduce lens fibrosis.

## Conclusions

In conclusion, we identified ARG1 as a profibrotic factor in lens fibrosis. Knockdown of ARG1 or pharmacological blockade of the ARG1 pathway effectively decreased collagen 1A1, fibronectin, and vimentin expression and cell migration. We believe that ARG1 promotes the production of collagen 1A1 by directly activating the arginase pathway and leads to lens fibrosis by reducing NO production and increasing superoxide levels, providing a new mechanism for the prevention and treatment of fibrotic cataracts.

## Data Availability

All other data supporting the findings of this study are available from the corresponding authors upon reasonable request.

## Abbreviations

ARG1: Arginase-1
EMT: Epithelial-to-mesenchymal transition
ASC: Anterior subcapsular cataract
PCO: Posterior capsular opacification
HLECs: Human lens epithelial cells
siARG1: ARG1-targeted siRNA
siNC: Negative control siRNA
RT–qPCR: Quantitative real-time polymerase chain reaction
P5C: Pyrroline-5- carboxylate
P5CR: Pyrroline-5- carboxylate reductase
PRODH: Proline dehydrogenase
ODC: Ornithine decarboxylase
OAT: Ornithine aminotransferase
NO: Nitric oxide
α-SMA: Alpha-smooth muscle actin
ZO-1: Zonula occludens-1
collagen 1A1: Collagen type I alpha1 chain
LECs: Lens epithelial cells
non-ASC: Nonanterior subcapsular cataract
IOL: Intraocular lens
Nd:YAG: Neodymium-doped yttrium aluminium garnet
TGF-β2: Transforming growth factor beta-2
COL4: Collagen type IV
ARVO: Association for Research in Vision and Ophthalmology
H&E: Haematoxylin and eosin
nor-NOHA: Nω-hydroxy-nor-L-arginine monoacetate
NOS: Nitric oxide synthase
iNOS: Inducible nitric oxide synthase
ROS: Reactive oxygen species
GAPDH: Glyceraldehyde-3-phosphate dehydrogenase
